# Analyzing EEG data during opium addiction treatment using a fuzzy logic-based machine learning model

**DOI:** 10.3389/fpsyt.2025.1635933

**Published:** 2025-11-03

**Authors:** Elnaz DehAbadi, Fateme Ayşin Anka, Fateme Vafaei, Hossein Lanjanian, Sajjad Nematzadeh, Mahsa Torkamanian-Afshar, Nazanin Aghahosseinzargar, Farzad Kiani, Peyman Hassani-Abharian

**Affiliations:** ^1^ Garmsar Branch, Islamic Azad University, Garmsar, Iran; ^2^ Political Sciences and Public Administration Department, Faculty of Economics, Administrative and Social Sciences, Istinye University, Istanbul, Türkiye; ^3^ Department of Biomedical Engineering, Shahed University, Tehran, Iran; ^4^ Basir Eye Health Research Center, Iran University of Medical Sciences, Tehran, Iran; ^5^ Cellular and Molecular Endocrine Research Center, Research Institute for Endocrine Molecular Biology, Research Institute for Endocrine Sciences, Shahid Beheshti University of Medical Sciences, Tehran, Iran; ^6^ Software Engineering Department, Engineering Faculty, Istanbul Topkapi University, Istanbul, Türkiye; ^7^ Computer Engineering Department, Faculty of Engineering, Fatih Sultan Mehmet Vakif University, Istanbul, Türkiye; ^8^ Data Science Application and Research Center, Fatih Sultan Mehmet Vakif University, Istanbul, Türkiye; ^9^ Department of Cognitive Psychology and Rehabilitation, Institute for Cognitive Science Studies (IRICSS), Tehran, Iran

**Keywords:** EEG data analysis, fuzzy logic, neural activity patterns, opium addiction, substance abuse treatment

## Abstract

**Background:**

Reliable noninvasive tools for assessing substance abuse treatment and predicting outcomes remain a challenge. We believe EEG-derived complexity measures may have a direct link to clinical diagnosis. To this aim, our study involved a psychological investigation of four groups of current and former male opium addicts. Furthermore, we propose a machine learning (ML) model incorporating fuzzy logic to analyze EEG data and identify neural complexity changes associated with opium addiction.

**Method:**

Male participants were categorized into four groups: active addicts, those with less than three days of treatment, those treated for over two weeks, and healthy controls. Psychological assessments evaluate mental health and addiction status. EEG data were collected using standardized electrode placement, preprocessed to remove noise, and analyzed using the Higuchi Fractal Dimension(HFD) to quantify neural complexity. Feature selection methods and ML classifiers were applied to identify key patterns distinguishing addiction stages.

**Results:**

Distress levels varied significantly across groups and persisted post-quitting. Addicts exhibited poorer general health than controls, though treatment led to improvements. Significant differences in neural complexity were observed in brain regions linked to attention, memory, and executive function. The ML model effectively classified addiction stages based on EEG-derived features.

**Conclusion:**

This study demonstrates the potential of ML and fuzzy logic in assessing addiction-related neural dynamics, offering insights into opioid addiction’s pathophysiology. The findings highlight the promise of brainwave-based biomarkers for personalized addiction diagnosis and treatment monitoring.

## Graphical Abstract

The flowchart illustrates the analysis of EEG data during opium addiction treatment using fuzzy logic-based machine learning. It includes the collection of data from opioid-addicted and healthy males, psychological evaluations, EEG data analysis with noise removal, and feature extraction. A fuzzy logic machine learning model is then developed for treatment management, which identifies levels of distress and differences in the complexity of neural activity.

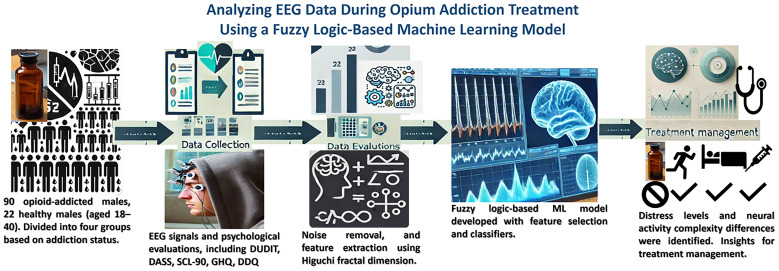

## 1 Introduction

Addiction can be defined as the loss of control over drug use or the compulsive seeking and use of drugs despite adverse consequences. It is a neuropsychiatric disorder caused by substance abuse that is strongly influenced by a person’s genetic structure and the psychological and social context in which drug use occurs. The disease cycle of addiction mainly results from dopaminergic dysfunction, particularly in dopamine (DA) secreted from the mesencephalic ventral tegmentum area (VTA) to the nucleus accumbens (NAcc), prefrontal cortex (PFC), and amygdala. Substances of abuse affect the same neural circuitry as primary biological rewards such as food, water, and sex, leading to substance dependence (1, 2). The understanding of addiction has evolved, from being viewed as a moral condition in DSM-II to being considered more based on psychobiological constructs in DSM-III and beyond. DSM-IV added cognitive factors to the contributing factors of addiction, while DSM-V focused on the psychological changes caused by substance abuse that lead to cognitive impairment (3). Consumed substances have different effects on neural processes, and substance use can lead to increased dopamine consumption in the collecting systems, which becomes an important element in goal-directed behavior and can ultimately result in substance dependence (2).

Nonlinear dynamics methods are widely used to analyze neurophysiological data due to the brain’s inherent complexity, which spans multiple spatial and temporal scales. Higuchi Fractal Dimension (HFD) specifically quantifies the self-similarity and complexity of neural oscillations, offering insights into the balance between stochasticity and determinism in brain dynamics. In the context of addiction, altered neural complexity may reflect disruptions in cognitive control, reward processing, and executive function. Various complexity measures have been developed, including those based on random fractal theory, information theory, and chaos theory. Gao et al. distinguish between chaos and random phenomena and found that the variations of complexity measures with time are either similar or reciprocal in their study on the relations among different complexity measures for EEG (4, 5).

Emotion recognition and addiction detection using electroencephalogram (EEG) signals have garnered increasing interest due to their potential applications in mental health monitoring, affective computing, and neurorehabilitation (6–8). Among various techniques applied to analyze EEG signals, fuzzy logic has emerged as a powerful mathematical framework to manage uncertainty and imprecision. Its inherent ability to model nonlinear and complex systems makes it highly suitable for EEG analysis, which often involves noisy, nonstationary, and high-dimensional data (9–12).

Fuzzy logic has been employed in diverse EEG-related domains, such as brain-computer interfaces (BCIs), neurological disorder diagnosis, and cognitive state assessment. For instance, studies have demonstrated the efficacy of fuzzy logic in classifying visual perception-related EEG signals and diagnosing epilepsy through fuzzy expert systems (13–16). The flexibility of fuzzy set theory has also enhanced feature processing by enabling partial membership-based selection, which improves the robustness of classification tasks. Tools like partition generator functions have been leveraged to filter and transform EEG feature vectors into sparse representations, particularly valuable in multi-instance data scenarios. The Partition generator function can be used in feature processing methods that are based on fuzzy set theory. These methods use the idea of partial set membership to identify relevant features that have high membership degrees for a given class or target variable. By using fuzzy set theory in feature selection, these methods can provide more flexible and accurate results than traditional binary-based methods. This filter supports multi-instance data and can be applied to a given dataset and features for any partition generator to obtain these filtered vectors for all instances. As a result of this, filtered instances are composed of the relevant values and the class attribute (if set in the input data) and rendered as sparse instances (14, 15).

Recent advancements in BCI technologies have further facilitated the integration of fuzzy logic with machine learning. Notably, Dhara et al. (2023) introduced a fuzzy ensemble-based deep learning model that attained over 97% accuracy on the DEAP dataset and 95% on AMIGOS, showcasing the synergy between fuzzy logic and deep learning in refining model predictions (11). In parallel, deep learning has gained significant momentum in EEG-based emotion recognition. Hassouneh et al. (2020) developed a real-time system combining facial expressions and EEG signals using machine learning and neural networks, highlighting the feasibility of accurate multimodal emotion classification (17). A comprehensive review by Chutia and Baruah (2024) emphasized the impact of convolutional and recurrent neural networks in capturing spatiotemporal dynamics of EEG data (6). Similarly, Khare et al. (2024) systematically reviewed a decade of emotion recognition studies, underlining the increasing shift toward hybrid models that integrate EEG with other physiological signals for improved contextualization (7). This trend is echoed in the review by Computers in Biology and Medicine (2023), which notes the growing importance of transfer learning, attention mechanisms, and multimodal fusion techniques in advancing EEG-based emotion detection (8).

Beyond emotion recognition, fuzzy logic and EEG complexity metrics have also proven useful in addiction research. For example, Marvi et al. (2023) applied recurrence quantification analysis and entropy indices to distinguish multidrug users from healthy individuals with 90% accuracy using support vector machines (18). Zhou et al. (2024) further extended these findings by employing wavelet-transformed P300 components and BiLSTM networks to detect methamphetamine abuse with 83.85% accuracy (19). Additionally, Crane et al. (2021) revealed altered neural reward processing in cannabis users via EEG event-related potentials, suggesting that EEG-based measures may serve as biomarkers for addiction-related dysfunctions (20). Finally, Hosseini et al. examined the effects of computer gaming on brain function, offering insights using quantitative EEG (QEEG) complexity analysis (21).

Collectively, these findings confirm that fuzzy logic approaches—whether standalone or hybridized with deep learning—and EEG-based signal complexity analysis substantially enhance both the accuracy and interpretability of brain activity monitoring in emotional and addiction-related research domains.

As mentioned previously, despite the widespread use of EEG in clinical neuroscience, there remains a critical need for reliable, noninvasive tools to assess substance addiction and monitor treatment progression. We introduce a novel EEG dataset comprising recordings from four distinct groups of male participants: active opium addicts, individuals undergoing early treatment (≤3 days), individuals in extended treatment (>2 weeks), and healthy controls. EEG signals were recorded from frontal and parietal regions, preprocessed to remove artifacts, and analyzed using the Higuchi Fractal Dimension to quantify EEG complexity. Additionally, fuzzy logic-enhanced machine learning model was applied to classify subjects based on their addiction stage. Addicted individuals exhibited reduced EEG complexity in regions associated with attention, memory, and executive function. These differences were partially reversed in long-term treated subjects. Fuzzy approach to HFD features of EEG complexity led to high classification accuracy across groups. This study advances the field by addressing limitations in previous works, refining EEG-based complexity analysis with fuzzy logic, and moving toward practical applications in addiction diagnosis and treatment monitoring.

## 2 Materials and methods

### 2.1 Participant selection

The research sample consisted of male participants selected from the Ahang De-Addiction Institute, a treatment center located in the 15th district of Tehran. Established in 1992, the institute provides addiction treatment services. Ethical authorization for research was received through letter No. IR.UT.IRICSS.REC.1403.021 from the Ethics Committee of the Institute for Cognitive Science Studies on March 12, 2024.

To reduce confounding effects and obtain a homogeneous sample, we restricted recruitment to male participants aged 18–40 years. This decision was based on evidence that EEG signals and brain complexity measures are influenced by both sex and age, with prior studies showing clear sex-related differences in brain activity and addiction patterns, as well as age-dependent changes in EEG complexity. Epidemiological surveys in Iran further support this choice, indicating that more than 90% of substance users are male and that opium use is most prevalent in young adults, with a mean age of initiation around 22 years and mean current ages in the early 30s (22, 23). While these studies are relatively old and addiction trends may have evolved, our clinical experience with referrals to addiction treatment centers continues to align with these statistics. Therefore, in this study we focused on 18–40-year-old males to capture the demographic group at highest risk, while minimizing variability unrelated to addiction status.

The participants were categorized into four groups based on their addiction status and treatment abstinence period. The first group comprised actively addicted individuals who had not yet started the treatment process. The second group consisted of individuals with less than three days of treatment. The third group included individuals who had undergone treatment for more than two weeks. The fourth group consisted of healthy individuals with no history of drug use.

The research was conducted over four consecutive days at a camp, with a psychologist from the research team present. Written consent was obtained from each participant before the study. The initial assessment process involved collecting a range of information, including basic demographics, substance abuse history (first-time drug use, duration of drug use, primary substance used, and expenditure on drugs), treatment history (number of quit attempts, longest period of abstinence, participation in Narcotics Anonymous meetings), history of risky behaviors (injection drug use, risky sexual behaviors, involvement with drug dealers, physical conflicts), medical and psychiatric information (chronic medical conditions, physical problems, depression, anxiety, hallucinations, delusions, suicide attempts or self-mutilation, history of mental health hospitalizations, HIV status), and family and social status (living conditions, employment status, monthly income, family history of addiction, number of children in the family, family’s emotional support).

Participants for the recently quit group and the control group were selected through the quit addiction camp and direct interviews. The active user group was recruited in collaboration with Ahang addiction treatment clinics, and EEG registrations were conducted with the consent of the patients. The study included 90 individuals undergoing treatment of opioid addiction and 22 healthy individuals with no history of drug abuse. All participants were males aged between 18 and 40 years. Opioid-addicted individuals were recruited from the AHANG drug rehabilitation center, including both active users and those who had recently sought treatment at the center. Psychological tests, such as the Drug Use Disorders Identification Test (DUDIT) and the Depression, Anxiety, and Stress Scale (DASS), were administered to all addicted individuals. Additionally, information regarding basic demographics, treatment history, history of risky behaviors, history of drug abuse, medical status, and family and social support was collected for the addicted group.

### 2.2 Psychological tests

Psychological assessments were conducted to evaluate the mental health and addiction status of participants. For the healthy group, a clinical interview, SCL-90 questionnaire, and urine test were administered to confirm mental health and the absence of addiction. The SCL-90 is a widely used psychiatric tool consisting of 90 items scored on a 5-point scale, assessing nine symptom dimensions including somatization, obsessive-compulsive behavior, interpersonal sensitivity, depression, anxiety, hostility, phobic anxiety, paranoid ideation, and psychoticism. In Iran, the SCL-90 has been standardized and validated in multiple studies.

The following psychological questionnaires were completed for each candidate:

Depression, Anxiety, and Stress Scale (DASS): The DASS questionnaire consists of three subscales designed to measure negative emotional states related to depression, anxiety, and tension. Each subscale contains 7 items that assess various aspects of these emotional states. From these 21 items, 8 items are related to depression, 7 items are related to anxiety and 6 items are related to stress.

General Health Questionnaire (GHQ): The GHQ is a self-administered test used to investigate non-psychotic disorders. It is a screening tool to identify individuals experiencing acute conditions or disturbances in functioning (24).

Desires for Drug Questionnaire (DDQ): The DDQ assesses craving for drugs and consists of 13 questions that measure three main craving components: desire and intention to use drugs, negative reinforcement, and control.

Obsessive Compulsive Drug Use Scale (OCDUS): The OCDUS questionnaire measures three components related to heroin use: heroin thoughts and interference, intention to use heroin and control of consumption and resistance against thoughts and decisions to use heroin.

Each questionnaire was completed using a Likert-scale answer sheet, with participants rating their experiences or feelings based on the provided options (25).

Note: The original versions of the DASS (26, 27), DDQ and OCDUS (28) questionnaires were modified to improve internal consistency by including additional questions.

### 2.3 EEG recording and data gathering

**Figure 1:**
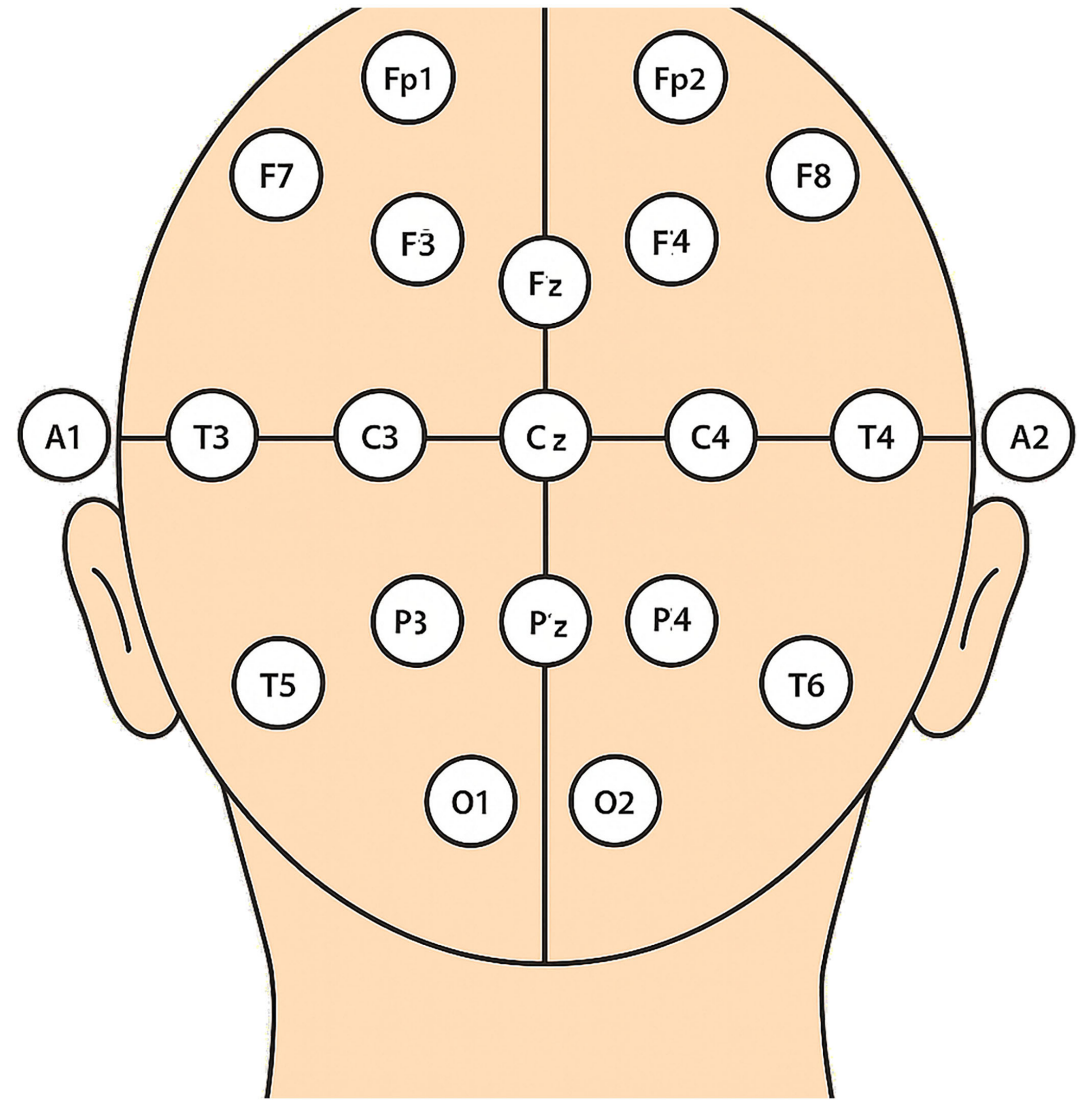
Electrode positions for the 19-channel EEG apparatus. EEG data were collected using a standardized 19-channel electrode placement scheme with the MEDICOM MTD device, following the international 10–20 system. The electrode locations included Fp1, Fp2, F7, F3, Fz, F4, F8, T3, C3, Cz, C4, T4, T5, P3, Pz, P4, T6, O1, and O2, using a Linked Ears reference.

EEG data was collected from participants using a standardized electrode placement scheme. The EEG signals were obtained from 19 active electrodes using the MEDICOM MTD device, following the standard 10–20 system electrode placement. The electrode locations included Fp1, Fp2, F7, F3, Fz, F4, F8, T3, C3, Cz, C4, T4, T5, P3, Pz, P4, T6, O1, and O2; using a Linked Ears reference. The sampling frequency of the EEG signals was 250 Hz. In line with our previous studies and to keep the approach simple and decrease the computation steps, we calculated the HFD for the total EEG signals (without focusing on any specific frequency band) was calculated (29). Except for the addict group, we recorded two EEG signals in closed eye and one signal in open eye states (**Figure 1**).

### 2.4 Signal preprocessing and data cleaning

Signal preprocessing was conducted on the recordings obtained from all 19 channels for each participant using EEGLAB v.2019. The following steps were followed:

- Artifact detection and removal: EEGLAB’s “Remove baseline” and “Reject data using Clean Raw data and ASR” options were utilized to eliminate artifacts from the recorded EEG signals.
- Out-of-band noise removal: A Finite Impulse Response (FIR) filter was applied to filter the EEG time series within a range of 1–70 Hz, effectively removing noise outside this frequency range.
- EEG re-referencing: Common average referencing was employed, which involved calculating the average of all channels and using it as the reference for each channel.
- Line noise suppression: A notch filter at 50 Hz was utilized to suppress line noise interference.
- Repairing bad or missing channels: Any channels that were deemed bad or missing were repaired through interpolation, which involved replacing them with the average value of their neighboring channels.
- Independent Component Analysis (ICA): ICA was applied to detect components within each signal in every channel.
- Removal of undesired components: Components originating from undesired sources, such as electrocardiography (ECG) and electromyography (EMG), were removed to obtain artifact-free EEG signals.

These preprocessing steps enhance the quality of the EEG data and minimize the impact of artifacts and noise, ensuring more reliable and accurate analysis in subsequent stages of the study.

It is noteworthy that only closed-eye EEG segments were analyzed, as the open-eye recordings did not provide sufficiently reliable results. This approach minimizes the influence of ocular artifacts and ensures that the analyzed EEG signals reflect neural activity relevant to the study.

### 2.5 Higuchi algorithm and feature extraction

Feature extraction is the process of transforming original data to remove redundant or irrelevant information and producing a much smaller and more manageable data set of more discriminator variables. Fractal theory can be used to extract features from a series. The Higuchi Fractal Dimension (HFD) algorithm is a method for measuring the fractal dimension of discrete-time sequences. Here, fractal dimensions were extracted as features using the Higuchi Fractal Dimension (HFD) algorithm (30, 31) (21).

### 2.6 Partition membership method and feature processing

The fuzzy function maps the membership degree of an element for a given set to a real value in between [0,1]. There are some feature processing methods based on the Partition generator function that come from the fuzzy set idea. To calculate these feature vectors for all instances, WEKA’s PartitionMembershipFilter was employed, which can apply any partition generator to a given dataset. The extracted features were filtered through propositionalization and partitioning using the PartitionMembership filter. WEKA (Waikato Environment for Knowledge Analysis) (32, 33), a non-commercial and open-source data mining system was utilized for this purpose.

For more details on the mathematical principles and applications of Higuchi’s Fractal Dimension (HFD) and fuzzy logic in our study, please refer to **Supplementary File 1**, which provides an in-depth explanation of the methods and their integration into the analysis of EEG data.

### 2.7 Feature selection

We are interested in the feature subset containing the minimum number of features that contribute to accuracy the most (29). Here we used CorrelationAttributeEval, ChiSquaredAttributeEval, SignificanceAttributeEval, and PrincipalComponents as the attribute evaluator and “Ranker” as the search method to find the most important attribute in discrimination between different groups. For three methods, WEKA outputs are a ranked list of attributes; however, to determine the priority of features using PrincipalComponents, we follow these steps: 1- Computing the correlation matrix; 2- Obtaining the eigenvalues and eigenvectors; 3- Sorting eigenvalues; eigenvalues represent the amount of variance explained by each principal component. Higher eigenvalues indicate more important principal components. 4- Selecting first principal components; 5- Determining feature importance; The coefficients within the eigenvectors indicate the importance of the original features in the respective principal component. Larger coefficients suggest higher importance (34).

### 2.8 Classification

Finally, supervised machine-learning classification using a Support Vector Machine (SVM) (35), MLPClassifier (Trains a multilayer perceptron with one hidden layer) and Multilayer Perceptron (A classifier that uses backpropagation to learn a multi-layer perceptron) were employed for feature selection. The calculations were done using WEKA default parameters.

### 2.9 Statistical analysis of features

A parallel analysis of machine learning (ML) was conducted to assess the statistical significance of Higuchi features using appropriate statistical methods. The objective was to identify which channels exhibited significantly different complexities between different groups. Firstly, the Kolmogorov-Smirnov test was employed to assess the normality of the data, revealing that the data satisfied the normality assumption (P > 0.05). Subsequently, the One-way ANOVA test was utilized to compare the four groups across 38 Higuchi variables. Tukey’s test is a single-step multiple comparison procedure and statistical test. It can be used to find means that are significantly different from each other. it compares all possible pairs of means and applies simultaneously to the set of all pairwise comparisons.

The same method was used to analyze the results of psychological assessments of participants. Here, we take together all participants who had undergone treatment (second and third groups).

## 3 Results

During the preprocessing steps, some records were detected as being corrupted or disturbed; therefore, the final numbers of analyzed participants of different groups were displayed in **Table 1**.

**Table 1:** The final numbers of analyzed participants.

Groups	EEG recording state	Number of participants	Number of EEG data after preprocessing	Age	Male
Addict	Eye close	19	19	40.5 ± 7.9	100%
	Eye open	19	19	40.5 ± 7.9	100%
First Group	Eye close	24	48	35.5 ± 6.6	100%
	Eye open	24	24	35.5 ± 6.6	100%
Second Group	Eye close	23	46	38. ± 9.2	100%
	Eye open	23	23	38. ± 9.2	100%
Normal	Eye close	20	36	37.1 ± 3.8	100%
	Eye open	20	18	37.1 ± 3.8	100%

### 3.1 Psychological tests

The results of the psychological assessments of participants are presented in **Table 2** and **Supplementary Table S1**. Here, we take together all participants who had undergone treatment (second and third groups).

**Table 2:** Results of the psychological assessments of the participants. Here, we take together all participants who had undergone treatment (second and third groups).

Description		Healthy group	Control (actively addicted) group	All participants who had undergone treatment	P value
		Mean ± SD			
DASS	Depression	4.18 ± 5.14	11.67 ± 6.41	12.3 ± 5.93	0.001
	Anxiety	3.76 ± 3.36	9.29 ± 5.27	11.61 ± 5.19	0.001
	Stress	6.53 ± 5.17	12.9 ± 5.77	14.65 ± 7.3	0.001
	Total	14.47 ± 12.07	33.86 ± 16.33	38.48 ± 16.14	0.001
	Number	17	21	23	–
GHQ	Physical	4.17 ± 2.6	4.76 ± 4.36	8.26 ± 4.26	0.002
	Sleep Anxiety	5.17 ± 3.49	6.19 ± 3.5	9.3 ± 4.99	0.005
	Social Interaction	7.17 ± 3.94	7 ± 4.99	8.57 ± 3.38	0.396
	Depression	3 ± 2.09	6.71 ± 5.15	6.78 ± 4.9	0.013
	Total	19.61 ± 10.16	24.67 ± 14.04	32.91 ± 13.51	0.006
	Number	18	21	23	–
OCDUS	Craving For Consumption And Mental Occupation Of Substances	–	9 ± 5.07	11.09 ± 4.59	0.159
	The Impact Of Drug Use On The Work And Life Of Consumers		4.24 ± 3	6.91 ± 2.61	0.003
	Motivation And Excitement Of Violation Of Control		8.19 ± 3.83	12.83 ± 4.48	0.001
	Resistance To Drug Use		4.05 ± 2.44	4.7 ± 2.22	0.362
	Total		25.1 ± 12.61	35.91 ± 11.9	0.006
	Number		21	23	–
DDQ	Craving And Intention To Consume		13.67 ± 5.79	17.13 ± 13.33	0.278
	Negative Reinforcement		9.19 ± 6.24	10.35 ± 7.29	0.576
	Consumption Control		3.86 ± 3.18	2.61 ± 1.53	0.1
	Total		26.33 ± 9.87	30.09 ± 20.17	0.444
	Number		21	23	–

For more details on the psychological assessments of the participants, please refer to **Supplementary File 2**.

### 3.2 Classification

In this study, we utilized HFD algorithms to convert the series of EEG data into scalar values, generating a set of features specific to each participant. The HFD algorithms processed the signals from each EEG channel, resulting in a total of 38 features for every participant. These features were derived from EEG channels, with pairs like Higu_1 and Higu_2 originating from channel 1, and the third and fourth Higuchi features originating from channel 2.

Initially, the machine learning methods struggled to distinguish between different classes without applying the fuzzy approach. To improve classification performance, we utilized the Partition Membership method in WEKA to filter and combine the extracted features. We then conducted classification experiments on both the raw (unprocessed) and filtered feature sets using three classification methods: MLPClassifier, LibSVM, and MultilayerPerceptron. This comparison allowed us to evaluate whether feature selection and transformation via Partition Membership improved classification accuracy and model robustness in differentiating addiction stages.

To evaluate the performance, we considered four distinct groups and examined a total of 11 combinations. These combinations included a category that encompassed all groups, four categories representing each group, one category where a single group was compared against a merged group of the remaining three, and six categories involving pairwise comparisons between groups. Before using the Partition Membership method in WEKA without a fuzzy approach, the machine learning methods failed to distinguish between different classes (data has been not shown). The results obtained from fuzzy analyses are presented in **Table 3**.

**Table 3:** The obtained results from the classifiers.

Groups	Instances	Classifiers	TP rate	FP rate	Precision	Recall	F-measure	MCC	ROC Area	PRC area	Class	Confusion matrix			
												a	b	c	d
All	114	Mlp	0.79	0.04	0.79	0.79	0.79	0.75	0.90	0.86	a = addict	15	1	2	1
			0.90	0.05	0.87	0.90	0.88	0.84	0.93	0.78	b = Second	1	26	1	1
			0.90	0.06	0.85	0.90	0.88	0.83	0.95	0.82	c = first	2	0	28	1
			0.83	0.04	0.91	0.83	0.87	0.81	0.95	0.84	d = normal	1	3	2	29
			0.86	0.05	0.86	0.86	0.86	0.81	0.93	0.82	Weighted Avg				
Addict vs Others	114	Mlp	0.11	0.11	0.17	0.11	0.13	0.00	0.48	0.22	a = addict	2	17		
			0.90	0.90	0.83	0.90	0.86	0.00	0.48	0.81	b = Others	10	85		
			0.76	0.76	0.72	0.76	0.74	0.00	0.48	0.71	Weighted Avg				
First vs Addict	50	Mlp	0.95	0.03	0.95	0.95	0.95	0.92	0.92	0.88	a = addict	18	1		
			0.97	0.05	0.97	0.97	0.97	0.92	0.92	0.90	b = first	1	30		
			0.96	0.05	0.96	0.96	0.96	0.92	0.92	0.89	Weighted Avg				
First vs Others	114	Mlp	0.98	0.19	0.93	0.98	0.95	0.82	0.87	0.91	a = Others	81	2		
			0.81	0.02	0.93	0.81	0.86	0.82	0.87	0.76	b = first	6	25		
			0.93	0.15	0.93	0.93	0.93	0.82	0.87	0.87	Weighted Avg				
First vs Second	60	Mlp	0.48	0.03	0.93	0.48	0.64	0.52	0.75	0.73	a = Second	14	15		
			0.97	0.52	0.67	0.97	0.79	0.52	0.75	0.72	b = first	1	30		
			0.73	0.28	0.80	0.73	0.72	0.52	0.75	0.73	Weighted Avg				
Normal vs Addict	54	Mlp	1.00	0.14	0.79	1.00	0.88	0.82	0.95	0.90	a = addict	19	0		
			0.86	0.00	1.00	0.86	0.92	0.82	0.95	0.97	b = normal	5	30		
			0.91	0.05	0.93	0.91	0.91	0.82	0.95	0.95	Weighted Avg				
Normal vs First	66	LibSVM	0.84	0.06	0.93	0.84	0.88	0.79	0.89	0.86	a = first	26	5		
			0.94	0.16	0.87	0.94	0.90	0.79	0.89	0.85	b = normal	2	33		
			0.89	0.11	0.90	0.89	0.89	0.79	0.89	0.85	Weighted Avg				
Normal vs Others	114	Mlp	0.99	0.03	0.99	0.99	0.99	0.96	0.98	0.99	a = others	78	1		
			0.97	0.01	0.97	0.97	0.97	0.96	0.98	0.91	b = normal	1	34		
			0.98	0.02	0.98	0.98	0.98	0.96	0.98	0.97	Weighted Avg				
Normal vs Second	64	Mlp	0.97	0.09	0.90	0.97	0.93	0.88	0.94	0.86	a = Second	28	1		
			0.91	0.03	0.97	0.91	0.94	0.88	0.94	0.97	b = normal	3	32		
			0.94	0.06	0.94	0.94	0.94	0.88	0.94	0.92	Weighted Avg				
Second vs Addict	48	LibSVM	0.90	0.07	0.90	0.90	0.90	0.83	0.91	0.84	a = addict	17	2		
			0.93	0.11	0.93	0.93	0.93	0.83	0.91	0.91	b = Second	2	27		
			0.92	0.09	0.92	0.92	0.92	0.83	0.91	0.88	Weighted Avg				
Second vs Others	114	Mlp	0.74	0.79	0.73	0.74	0.74	-0.05	0.48	0.76	a = Others	62	23		
			0.21	0.26	0.21	0.21	0.21	-0.05	0.48	0.27	b = Second	21	8		
			0.61	0.66	0.60	0.61	0.60	-0.05	0.48	0.63	Weighted Avg				
Normal vs First	66	Mlp	0.58	0.34	0.60	0.58	0.59	0.24	0.63	0.62	a = first	18	13		
			0.66	0.42	0.64	0.66	0.65	0.24	0.63	0.61	b = normal	12	23		
			0.62	0.38	0.62	0.62	0.62	0.24	0.63	0.62	Weighted Avg				
Second vs Addict	48	Mlp	0.74	0.00	1.00	0.74	0.85	0.79	0.98	0.97	a = addict	14	5		
			1.00	0.26	0.85	1.00	0.92	0.79	0.98	0.99	b = Second	0	29		
			0.90	0.16	0.91	0.90	0.89	0.79	0.98	0.98	Weighted Avg				

It is noteworthy that the MultilayerPerceptron method yielded the most favorable outcomes for the majority of categories when the filtered attributes were utilized. However, in two categories, the Partition Membership filter only produced one partition and failed to effectively classify the participants. Additionally, in two categories, the LibSVM method outperformed the MultilayerPerceptron method. The results obtained from the MultilayerPerceptron method are also provided for these two categories.

### 3.3 Feature priority

To evaluate the effectiveness of the feature processing, the study assessed the output of four different feature selection methods. CorrelationAttributeEval, Principal Components, ChiSquaredAttributeEval, and SignificanceAttributeEval are the employed methods. However, the last two methods did not find any difference between the features except two; higu_29 for First *vs* Others and higu_9 Normal *vs* Second; interestingly, these attributes were also proposed by the CorrelationAttributeEva methods. For the other two methods, we select the 5 high-ranked attributes and present them in **Supplementary Table S2**. By analyzing the eigenvalues and eigenvectors, you can identify the most influential features in your dataset. Features with high coefficients in the principal components associated with large eigenvalues contribute significantly to the variability and can be considered high-priority features. For more details on the Feature priority, please refer to **Supplementary File 2**.

The statistical analysis revealed significant differences among the four groups in terms of Higuchi variables. Specifically, the variables higu_7, higu_9, higu_11, and higu_29 exhibited statistically significant variations (P value < 0.05). These variables were derived from channels 4, 5, 6, and 15, respectively. To further investigate the differences within the groups, *post hoc* analysis was performed using Tukey’s test. The results of the *post hoc* analysis are summarized in **Supplementary Table S3**, which presents the variables that showed a statistically significant difference between at least two different groups.

**Table 3** presents classification performance across the analyzed groups. Each comparison is explicitly labeled, e.g., “First Group *vs*. Second Group,” to avoid ambiguity. The table is formatted in the standard three-line style (top line, header-body separator, and bottom line) for clarity. Key features contributing to group differentiation are mapped to their corresponding EEG channels, and anatomical regions (e.g., prefrontal cortex, limbic system) are indicated. This allows linking changes in neural complexity to cognitive processes associated with addiction, such as executive control, decision-making, and reward processing.

## 4 Discussion

This study aims to propose a machine learning (ML) model based on fuzzy logic for analyzing EEG data in order to enhance the understanding of the biological mechanisms underlying opium addiction and enable personalized diagnostics based on individual neural activity patterns. We analyzed EEG data from four groups based on their addiction status and treatment abstinence period by using of extracted complexity features that serve as indicators of neural dynamics in the brain. Classification experiments using different methods and attribute sets showed that MultilayerPerceptron function in combination with WEKA partition membership preprocessing resulted in outstanding results. All participants in this study were male and between 18–40 years of age. While this demographic restriction limits the generalizability of our findings to young adult male opioid users, it also provides an advantage in terms of methodological rigor. By studying a relatively homogeneous group, we were able to partially offset the limitations of a small sample size and reduce the influence of confounding factors such as sex, age, and ethnicity. This approach increases the internal validity of our findings, although future studies with larger and more diverse populations will be needed to confirm their broader applicability.

**Table 2** and **Supplementary Table S1** present the results of psychological assessments that were conducted to evaluate the mental health and addiction status of the participants.

The DASS questionnaire revealed that despite addiction treatment, the depression factor remained unchanged and did not differ between the control and addiction groups, but it decreased in the healthy group. In terms of anxiety, there was no significant difference between the recently quit group and the control group, but both differed significantly from the healthy group. The stress factor showed no significant difference between the recently quit group and the control group, but both significantly differed from the healthy group. Overall, the DASS questionnaire indicated that distress levels were significantly different between the groups, and even after quitting, the difference persisted.

Regarding the GHQ questionnaire, individuals who had recently quit exhibited higher levels of physical symptoms, which resolved immediately after quitting. The control group, who had quit more recently, showed similarities to the healthy group. The sleep factor also significantly differed between the recently quit group and both the control and healthy groups. Social interaction did not significantly differ between the three groups and did not change after quitting. The depression factor in the GHQ questionnaire aligned with the findings from the DASS questionnaire. In general, the total factors of the GHQ questionnaire demonstrated that addicted individuals had lower general health compared to healthy individuals. Treatment improved general health but still posed challenges compared to the healthy group.

Regarding the OCDUS questionnaire, cravings, and mental preoccupation did not show significance or differences between the recently quit group and the control group. However, the impact of drug use on work and life significantly differed between the recently quit group and the control group. Significant differences were also found in motivation, excitement, and violation of control between the recently quit group and the control group, indicating that emotional experiences were more consciously regulated in different situations. There was no significant difference in resistance to drug use between the two groups. Overall, the OCDUS questionnaire showed a significant difference in factors between the two groups, indicating a decrease in the urge to consume in the past week for both compared groups. However, intention and desire to consume were not significant, and there was high variability in dispersion and standard deviation.

Finally, the DDQ questionnaire did not yield any significant differences between the two groups in all factors.

The fuzzy-rough feature selection method involves using fuzzy logic to calculate the relevance of each feature and then using rough set theory to select the most relevant features. The method was shown to outperform other feature selection methods in terms of accuracy and efficiency (36–38). However, here we used WEKA partition membership as a filter based on fuzzy logic. It is clear from **Table 3** that Classification experiments using different methods and attribute sets were conducted, revealing the varying performance of the MultilayerPerceptron, LibSVM, and MLPClassifier methods across different categories. In consistence with our previous finding (21), the performance of MultilayerPerceptron function in combination with WEKA partition membership preprocessing shows outstanding results (**Table 3**).

The pattern of brain function in people who are using can be different from those who are quitting and healthy people, also the function of the brain can change under the influence of the type of substance consumed (39) For example, by comparing the EEG of alcoholics with the normal control group, an increase in beta power and a decrease in alpha power, as well as a decrease in the ratio of delta to theta, were confirmed (40). Marijuana consumption also leads to instability of the alpha frequency band (41, 42). According to the EEG recorded from people who have recently quit heroin a decrease in alpha, an increase in beta and an increase in Delta and Theta have been encountered in the central areas (43, 44). As a result, the people in this research were divided into four groups, the Addicted group included people who had just visited and the treatment process had not yet started for them, in other words, they were addicted, the First group included people who had less than three days of treatment. The second group included people who had been treated for more than two weeks, and finally the Normal group, who were healthy people who had no history of drug use. Using fuzzy logic techniques, the extracted features were processed to develop an ML model. Thus, we employed HFD algorithms to convert EEG data into scalar values, resulting in a set of features for each participant. These features were then filtered and combined using the Partition Membership method.

To evaluate the ability of different Higuchi variables(features) in discriminating different groups the output of four different feature selection methods is presented in **Supplementary Table S2**. Additionally, the study used a statistical analysis with the same aim (**Supplementary Table S3**). According to these results, we find out that different channels are responsible for the segregation of different groups.

The comparison between the Addict group and the other groups identified significant differences in Higuchi variables across channels 2, 4, 6, 8, 9, 10, 12, 16, and 17. These channels likely correspond to brain regions associated with addictive behaviors and related cognitive processes. Addiction involves complex interactions between several brain regions, including the prefrontal cortex, limbic system, and reward pathways. The observed differences in Higuchi variables within these channels may reflect altered neurophysiological activity linked to addictive tendencies.

The comparison of all groups against each other revealed significant differences in Higuchi variables across channels 2, 4, 5, 8, 9, 10, 16, 17, and 18. These channels potentially correspond to brain regions involved in various neurocognitive functions shared among the groups, such as attention, memory, and executive control. The observed differences in Higuchi variables within these channels suggest variations in the complexity of neural activity, reflecting the diversity of cognitive processes exhibited across the groups.

Comparisons of the First group with other groups revealed distinct sets of EEG channels exhibiting significant differences in Higuchi Fractal Dimension (HFD) variables, reflecting variations in neural complexity. For instance, when comparing the First group with the Addict group, channels 1, 4, 6, 9, 10, 15, 16, 17, and 19 showed significant differences, which may correspond to brain regions implicated in substance use disorders and associated cognitive impairments, such as executive control, reward processing, and attention regulation (45). Similarly, comparisons between the First group and the Other groups (channels 4, 5, 8, 9, 10, 15, 16, 17, and 19), and between the First and Second groups (channels 2, 3, 4, 5, 6, 13, 16, 17, and 19) revealed significant HFD differences, suggesting variations in neurophysiological activity that may underlie the unique cognitive and behavioral profiles of the First group (31).

Additional group comparisons also demonstrated channel-specific differences in HFD values: Normal *vs*. Addict (channels 2, 6, 9, 11, 12, 13, 17, 18), Normal *vs*. First (channels 3, 4, 5, 9, 11, 15, 17, 19), Normal *vs*. Others (channels 4, 8, 9, 10, 11, 16, 17, 18), Normal *vs*. Second (channels 2, 3, 4, 5, 8, 9, 11, 14, 18), Second *vs*. Addict (channels 2, 3, 4, 5, 8, 9, 10, 12, 16, 17), and Second *vs*. Others (channels 2, 3, 5, 8, 9, 10, 14, 16, 17, 18). These results indicate group-specific variations in neural complexity, potentially reflecting differential engagement of brain regions involved in addiction-related cognitive processes, including decision-making, inhibitory control, and reward sensitivity (46). Overall, these findings highlight the utility of HFD-based EEG analysis for capturing subtle neurophysiological differences across addiction stages and treatment groups.

The very high classification performance reported in **Table 3** (e.g., Normal *vs*. Others, F1 = 0.98; Normal *vs*. Addict, F1 = 0.91) should be interpreted with caution. Although we employed strict 10-fold cross-validation, evaluation on a held-out independent test set, and dimensionality reduction via the Partition Membership filter, the possibility of overfitting cannot be entirely excluded. One potential risk is that the model may have captured non-neural artifacts (e.g., head movements, muscle activity, or subtle recording differences) that coincidentally distinguished the groups. At the same time, the application of fuzzy logic to EEG feature processing constitutes a key methodological innovation of this study. Unlike traditional feature selection methods (e.g., correlation-based or chi-square) that operate on binary inclusion–exclusion rules, fuzzy logic allows for partial membership of features to classes, offering a more flexible and realistic way to address the inherent variability and uncertainty of EEG data. This approach not only enhances classification accuracy by retaining weak but informative signals that would otherwise be discarded under strict thresholding, but also improves interpretability: membership degrees provide an intuitive measure of the relative importance of each feature for group separation. Furthermore, the Partition Membership filter generates sparse feature representations that effectively reduce dimensionality while preserving subtle discriminative patterns, thereby improving robustness to noise. Taken together, these theoretical advantages (**Supplementary Table S1 of Supplementary File 1**) highlight fuzzy logic as a promising framework for uncovering latent neural patterns that may be overlooked by conventional methods, while also underscoring the need for careful validation to ensure that observed performance reflects genuine brain signal differences rather than overfitting artifacts. It is important to consider that the functional interpretations of these EEG channels and their relationships with neurocognitive and neurophysiological activities may require further investigation. Incorporating additional techniques, such as neuroimaging and comprehensive cognitive assessments, would contribute to a more comprehensive understanding of the brain regions involved and the specific neurocognitive and neurophysiological activities underlying the observed differences in Higuchi variables across these EEG channels. One of the main goals underlying this study is to focus on and interpret the issue from various dimensions, as it is a social problem that can expand to a large-scale social level. In this regard, as is evident in the study, an interdisciplinary study has been carried out by making use of both social sciences and engineering. As a result of the study, it would be a good idea and useful if the artificial intelligence-based model in this study could be connected to online tools that can evaluate the effectiveness of addiction treatment institutions and government spending. In this way, both public and private institutions can be managed effectively, and more accurate and rapid steps can be taken for patients. Therefore, policy development and implementation principles can be presented on the public side. The findings of the study provide important information for better understanding the pathophysiology of opioid addiction and personalizing individual diagnoses. This information can be used in the field of public administration to develop programs to combat substance addiction and to take and implement preventive measures. In particular, new substance addiction policies can be developed to increase the effectiveness of treatment programs and to take individual differences into account in addiction treatment. In addition, this study can make significant contributions for improving health services in public administration, increasing social welfare and security, and activating educational programs.

The second activity in the public administration dimension is the efficient management of health services. With the right plans for this, both the management of treatment centers and resource allocation and planning will become easier. As the third activity, education and awareness raising can be emphasized. In this regard, public awareness programs can be organized about the causes, effects, and treatment methods of addiction. The content of such training programs can be updated and made more effective in light of the findings of the study.

The last activity in public administration is social welfare and security. This may be among the issues that politicians need to deal with directly and put forward solutions. Of course, it may vary from country to country. In this context, the relationship between addiction and crime needs to be evaluated. Considering the impact of substance abuse on crime rates, effective management of addiction treatment can increase social safety. Public administration can develop effective policies for the rehabilitation and reintegration of individuals struggling with addiction. Social services and support programs can be developed to ensure the integration of individuals receiving addiction treatment into society. In this context, the results of the study can be used to increase the effectiveness of social service models used in the fight against addiction. Also, dependent people will not be excluded. Therefore, with these four basic activities in public administration, important steps will be taken towards the United Nations Sustainable Development Goals.

### 4.1 Limitation

The relatively small sample size of this study, particularly in relation to the number of pairwise (up to 11) and four-group comparisons, limits the statistical power to detect smaller effects. This may increase the likelihood of false negatives and, conversely, may also result in overestimation of significant effects. We also note that our study was designed as a proof-of-concept exploratory analysis to assess the feasibility of nonlinear EEG dynamics and fuzzy logic–based machine learning in the context of opioid addiction. We believe the promising preliminary results support further investigation in larger cohorts, and we have highlighted this as an important future direction.

### 4.2 Future directions

In the present study, we restricted our analyses to closed-eye resting-state EEG recordings. However, the nonlinear measures applied here—including Higuchi’s fractal dimension, entropy, and fuzzy entropy—have also been shown to be effective in task-based or functional EEG paradigms, where they capture task-specific brain dynamics and cognitive load (23, 47–51). This suggests that our framework could be readily extended to functional EEG experiments, such as during memory, attention, or decision-making tasks. Consequently, we propose that the Partition Membership filtering and fuzzy-based feature processing described in this work are suitable for task-based EEG analyses and could be used in follow-up studies to probe stimulus- or task-specific signatures of addiction. Exploring this avenue in future work may help to clarify whether the observed differences in brain complexity generalize beyond resting-state conditions and provide richer insights into the neurophysiological mechanisms under study.

In future studies, it would be valuable to extend our methodology beyond eyes-closed resting-state EEG to include task-related or stimulus-evoked EEG paradigms, and to examine joint complexity with other physiological signals such as electrocardiography (ECG) or respiration. Prior work has shown that EEG and ECG complexity correlate via multiscale entropy (52) and that complexity synchronization across organ systems may reveal deeper insights into neurophysiological regulation (53).

## 5 Conclusion

This study presents a machine learning framework integrating fuzzy logic to analyze EEG data, offering an objective tool for assessing addiction severity and treatment progress. By extracting Higuchi Fractal Dimension features, refining them with the Partition Membership method, and applying classifiers, we identified Multilayer Perceptron as the most effective model for distinguishing addiction stages. Traditional addiction assessments rely heavily on self-reported data, which can be incomplete, biased, or unreliable. The challenge of obtaining accurate treatment histories underscores the value of our approach: EEG-based complexity measures combined with fuzzy logic provide an objective and data-driven complement to traditional demographic and clinical assessments, helping to reduce dependence on self-explanation by addicted individuals. The findings highlight persistent brain function disruptions even after treatment initiation, emphasizing the need for objective, brain-based biomarkers. Overall, this work demonstrates the potential of AI-driven neurophysiological analysis to strengthen addiction research. Future efforts should prioritize broader validation and explore real-time monitoring applications, paving the way toward more personalized and effective treatment strategies.

## Data Availability

The raw data supporting the conclusions of this study can be found here: https://github.com/sajjad-nematzadeh/EEGOpium. Further inquiries can be directed to the corresponding author/s.
