# Supplementary material for: Analyzing EEG data during opium addiction treatment using a fuzzy logic-based machine learning model
Source: Front Psychiatry. 2025 Nov 3;16:1635933. doi: 10.3389/fpsyt.2025.1635933 (PMC12620379; doi:10.3389/fpsyt.2025.1635933)
Supplement: Supplementary file 1 [file SupplementaryFile1.docx]

**Supplementary material**

**Higuchi’s Fractal Dimension (HFD)**

Higuchi’s Fractal Dimension (HFD) is a mathematical approach for quantifying the complexity of time-series data, such as EEG signals. It measures the fractal dimension of a signal, capturing its self-similarity and irregularity across different time scales. Unlike traditional linear methods, HFD provides a more detailed representation of signal dynamics by assessing how the waveform fills space over varying segment lengths.

By computing HFD, we can analyze the intricate fluctuations in EEG signals, offering insights into neural activity patterns. Higher HFD values indicate greater signal complexity, often associated with healthier or more adaptive brain function, while lower values suggest reduced complexity, which can be linked to pathological states. This capability makes HFD particularly valuable for distinguishing between different neural states, such as addiction and recovery, by capturing subtle changes in temporal dynamics that conventional methods might overlook.

**Mathematical Formulation**

Given a time series *X= { x(1), x(2),...,x(N)},* the HFD is calculated as follows:

1. **Construct New Time Series**
   For each integer *k* (scale parameter), construct *k* new time series:

$X_{m}^{k}$*={x(m), x(m+k), x(m+2k), ..., x(m+⌊(N−m)/k⌋ k)}*

where m=1, 2, ..., k represents the starting point of the sequence.

1. **Compute the Length of Each Time Series**
   The length of each new sequence is given by:

$L_{m}$*(k)=*$\frac{1}{k}\sum_{i=1}^{\lfloor(N-m)/k\rfloor} \mid x(m+ik)-x(m+(i-1)k)\mid\frac{N-1}{\lfloor(N-m)/k\rfloor k}$

1. **Average Over All-Time Series**
   Compute the mean length over all sequences for a given kk:

*L(k)=*$\frac{1}{k}\sum_{m=1}^{k} L_{m}(k)$

1. **Fractal Dimension Estimation**
   Plot *log L(k)* versus *log(1/k)*, and estimate the fractal dimension **D** as the slope of the best-fit line:

$$D=-\frac{d logL(k)}{d log k}$$

This measure reflects the complexity of the EEG signal, with higher values indicating more irregular and complex neural activity.

**Fuzzy Logic-Based Classification**

Fuzzy logic is employed to manage uncertainty and imprecision in EEG data classification. Unlike traditional machine learning models that rely on hard decision boundaries, fuzzy logic allows for partial membership in multiple classes, offering a more flexible approach.

To evaluate the contribution of each feature (Higuchi Fractal Dimension, HFD) in distinguishing between target groups, we utilized fuzzy membership functions. These functions assign a membership value between 0 and 1, representing the degree of association between a feature and a classification group. Instead of rigid categorization, this approach enables a more nuanced differentiation based on predefined metrics such as variability and relative abundance. A membership value close to 1 signifies a strong association with a particular group (e.g., addicted), whereas values near 0 indicate minimal relevance.

Our fuzzy membership function incorporated two key aspects: (1) the variability in feature distributions across groups and (2) the relative value of each feature within the groups. Features displaying consistent differences between groups were assigned higher membership degrees, allowing us to capture even subtle variations. By leveraging fuzzy logic, we addressed the limitations of traditional threshold-based methods, which often struggle with overlapping distributions, thereby improving the robustness and interpretability of the classification process.

**Mathematical Principles**

A fuzzy system consists of **fuzzy sets, membership functions, fuzzy inference rules, and defuzzification**:

1. **Fuzzy Membership Functions**
   Each EEG feature *x* is assigned a membership value *μ(x)* in a fuzzy set.

A typical **Gaussian membership function** is:

μ(x)=$e^{\frac{{(x-a)}^{2}}{{2\sigma}^{2}}}$

where c is the center and σ controls the spread.

1. **Fuzzy Rule Base**
   A fuzzy inference system (FIS) applies **if-then rules** such as:

*IF* $x_{1}$*is Low AND* $x_{2}$ *is High, THEN Class = Addicted*

Each rule is evaluated using **fuzzy operators** (e.g., min, max):

*Rule Strength=*$min (\mu_{1}(x_{1}), (\mu_{2}(x_{2}))$

1. **Fuzzy Inference and Defuzzification**
   The final output is obtained by **defuzzification**, which converts fuzzy scores into a crisp value. A common method is the **center of gravity**:

y=$\frac{\sum_{i} \mu_{i}\left( x \right).c_{i}}{\sum_{i} \mu_{i}\left( x \right)}$

where cic_i represents the class labels (e.g., "Addicted" or "Healthy").

Fuzzy logic provides a flexible and interpretable classification framework, allowing EEG features to belong to multiple classes with different degrees of certainty.

**Comparison of traditional feature selection methods and fuzzy logic–based Partition Membership filtering**

| Aspect | Traditional Feature Selection (e.g., Correlation, Chi-Square, PCA) | Fuzzy Logic (Partition Membership Filter) |
| --- | --- | --- |
| Decision rule | Binary inclusion/exclusion of features based on thresholds or statistical tests | Allows **partial membership**, assigning a degree of relevance of each feature to each class |
| Handling of uncertainty | Limited ability to deal with noise and variability in EEG data | Explicitly models **fuzziness and uncertainty**, accommodating overlapping class boundaries |
| Information retention | May discard weak but informative features if they fail threshold | Preserves subtle discriminative patterns through weighted contributions |
| Output representation | Full feature vectors or reduced dimensions (e.g., principal components) | **Sparse representations** retaining only high-membership features, improving robustness |
| Interpretability | Focus on statistical ranking or variance explained | Membership degrees provide an **interpretable measure of feature–class relevance** |

**Pseudocode for PartitionMembershipFilter**

**Input:**

- Feature matrix X (n_samples × n_features)

- Class labels Y

- Parameters: fuzziness coefficient m, sparsity threshold λ

**Output:**

- Selected feature subset F_selected

**Algorithm:**

1. Initialize:

Compute initial feature relevance scores R(f) using correlation or mutual information with labels Y.

2. Fuzzy Partitioning:

For each feature f in X:

- Compute partial membership μ_f(c) of feature f to each class c

using fuzzy set membership:

μ_f(c) = 1 / Σ ( (||x_f - c|| / ||x_f - k||)^(2/(m-1)) ) over all classes k

𝑋= raw feature matrix.

𝜇_𝑓(𝑐)= fuzzy degree of membership of feature 𝑓 to class 𝑐, computed from 𝑋.

3. Sparse Representation:

- Construct membership matrix M where M[f, c] = μ_f(c)

- Apply sparse representation with threshold λ to remove redundant memberships:

If |M[f, c]| < λ → set M[f, c] = 0

4. Feature Partition Scoring:

For each feature f:

- Compute partition score S(f) = Σ_c (μ_f(c) * R(f))

- Normalize scores to [0,1]

5. Feature Selection:

- Rank all features by S(f)

- Select top-k features with highest scores

- Output as F_selected

End

**Description:**

🔹 The feature matrix 𝑋 contains the measured EEG features (entropy, power bands, nonlinear indices, etc.) for each subject/instance.

🔹 Fuzzy set theory & partial membership → in Step 2 of the pseudocode (μ_f(c) computation).

🔹 Partition generator function → the process of computing and structuring membership values (Steps 2–3).

🔹 The partial membership values μ_f(c) are derived quantities that you compute from 𝑋, based on fuzzy set theory.

Here’s the relationship:

1- Start with 𝑋∈𝑅 𝑛×𝑚, where 𝑛 = number of subjects/instances,𝑚= number of features.

2- For each feature 𝑓, you evaluate how strongly it belongs to a class 𝑐(e.g., addicted, recovering, control).

This is done via a membership function, e.g.:

𝜇_𝑓(𝑐)=(mean value of feature 𝑓 in class 𝑐) /(max mean across all classes)

or another fuzzy rule (triangular, Gaussian, etc.).

🔹 Sparse representations → Step 3 (apply sparse representation with threshold λ).

🔹 Multi-instance data support → mentioned in Step 3 and Step 5, since the filter works per instance and then aggregates.

🔹 Filtered instances with relevant values & class attribute → represented in Step 5 (output F_selected can be joined back with class labels to form sparse filtered instances).
