# Supplementary material for: Analyzing EEG data during opium addiction treatment using a fuzzy logic-based machine learning model
Source: Front Psychiatry. 2025 Nov 3;16:1635933. doi: 10.3389/fpsyt.2025.1635933 (PMC12620379; doi:10.3389/fpsyt.2025.1635933)
Supplement: Supplementary file 2 [file SupplementaryFile2.docx]

**Supplementary material**

**Table S1:** Results of Tukey test for comparing all possible pairs the psychological assessments of the participants. Here, we take together all participants who had undergone treatment (second and third groups)**.**

| Description | | | | P value | 95% Confidence Interval |  |
| --- | --- | --- | --- | --- | --- | --- |
|  |  |  |  |  | Lower Bound | Upper Bound |
| DASS | Depression | Healthy | Control (actively addicted) Group | 0.001 | -12.1191 | -2.8613 |
|  |  |  | All participants who had undergone treatment | 0.000 | -12.6658 | -3.5899 |
|  |  | Control (actively addicted) Group | Healthy | 0.001 | 2.8613 | 12.1191 |
|  |  |  | All participants who had undergone treatment | 0.932 | -4.9199 | 3.6446 |
|  |  | All participants who had undergone treatment | Healthy | 0.000 | 3.5899 | 12.6658 |
|  |  |  | Control (actively addicted) Group | 0.932 | -3.6446 | 4.9199 |
|  | Anxiety | Healthy | Control (actively addicted) Group Control (actively addicted) Group | 0.002 | -9.2800 | -1.7620 |
|  |  |  | All participants who had undergone treatment | 0.000 | -11.5292 | -4.1588 |
|  |  | Control (actively addicted) Group | Healthy | 0.002 | 1.7620 | 9.2800 |
|  |  |  | All participants who had undergone treatment | 0.251 | -5.8005 | 1.1545 |
|  |  | All participants who had undergone treatment | Healthy | 0.000 | 4.1588 | 11.5292 |
|  |  |  | Control (actively addicted) Group | 0.251 | -1.1545 | 5.8005 |
|  | Stress | Healthy | Control (actively addicted) Group | 0.008 | -11.2796 | -1.4711 |
|  |  |  | All participants who had undergone treatment | 0.000 | -12.9307 | -3.3148 |
|  |  | Control (actively addicted) Group | Healthy | 0.008 | 1.4711 | 11.2796 |
|  |  |  | All participants who had undergone treatment | 0.626 | -6.2844 | 2.7896 |
|  |  | All participants who had undergone treatment | Healthy | 0.000 | 3.3148 | 12.9307 |
|  |  |  | Control (actively addicted) Group | 0.626 | -2.7896 | 6.2844 |
|  | Total | Healthy | Control (actively addicted) Group | 0.001 | -31.3121 | -7.4610 |
|  |  |  | All participants who had undergone treatment | 0.000 | -35.6990 | -12.3164 |
|  |  | Control (actively addicted) Group | Healthy | 0.001 | 7.4610 | 31.3121 |
|  |  |  | All participants who had undergone treatment | 0.575 | -15.6536 | 6.4114 |
|  |  | All participants who had undergone treatment | Healthy | 0.000 | 12.3164 | 35.6990 |
|  |  |  | Control (actively addicted) Group | 0.575 | -6.4114 | 15.6536 |
| GHQ | Physical | Healthy | Control (actively addicted) Group | 0.883 | -3.5993 | 2.4088 |
|  |  |  | All participants who had undergone treatment | 0.004 | -7.0373 | -1.1511 |
|  |  | Control (actively addicted) Group | Healthy | 0.883 | -2.4088 | 3.5993 |
|  |  |  | All participants who had undergone treatment | 0.011 | -6.3217 | -0.6762 |
|  |  | All participants who had undergone treatment | Healthy | 0.004 | 1.1511 | 7.0373 |
|  |  |  | Control (actively addicted) Group | 0.011 | 0.6762 | 6.3217 |
|  | Sleep Anxiety | Healthy | Control (actively addicted) Group | 0.720 | -4.2041 | 2.1565 |
|  |  |  | All participants who had undergone treatment | 0.006 | -7.2535 | -1.0218 |
|  |  | Control (actively addicted) Group | Healthy | 0.720 | -2.1565 | 4.2041 |
|  |  |  | All participants who had undergone treatment | 0.039 | -6.1023 | -0.1255 |
|  |  | All participants who had undergone treatment | Healthy | 0.006 | 1.0218 | 7.2535 |
|  |  |  | Control (actively addicted) Group | 0.039 | 0.1255 | 6.1023 |
|  | Social Interaction | Healthy | Control (actively addicted) Group | 0.991 | -3.0351 | 3.3684 |
|  |  |  | All participants who had undergone treatment | 0.535 | -4.5354 | 1.7383 |
|  |  | Control (actively addicted) Group | Healthy | 0.991 | -3.3684 | 3.0351 |
|  |  |  | All participants who had undergone treatment | 0.428 | -4.5738 | 1.4433 |
|  |  | All participants who had undergone treatment | Healthy | 0.535 | -1.7383 | 4.5354 |
|  |  |  | Control (actively addicted) Group | 0.428 | -1.4433 | 4.5738 |
|  | Depression | Healthy | Control (actively addicted) Group | 0.028 | -7.0973 | -0.3313 |
|  |  |  | All participants who had undergone treatment | 0.022 | -7.0970 | -0.4682 |
|  |  | Control (actively addicted) Group | Healthy | 0.028 | 0.3313 | 7.0973 |
|  |  |  | All participants who had undergone treatment | 0.999 | -3.2472 | 3.1105 |
|  |  | All participants who had undergone treatment | Healthy | 0.022 | 0.4682 | 7.0970 |
|  |  |  | Control (actively addicted) Group | 0.999 | -3.1105 | 3.2472 |
|  | Total | Healthy | Control (actively addicted) Group | 0.442 | -14.9651 | 4.8540 |
|  |  |  | All participants who had undergone treatment | 0.005 | -23.0106 | -3.5932 |
|  |  | Control (actively addicted) Group | Healthy | 0.442 | -4.8540 | 14.9651 |
|  |  |  | All participants who had undergone treatment | 0.093 | -17.5579 | 1.0652 |
|  |  | All participants who had undergone treatment | Healthy | 0.005 | 3.5932 | 23.0106 |
|  |  |  | Control (actively addicted) Group | 0.093 | -1.0652 | 17.5579 |

**Table S2**: The attribute selection results.

| Groups | Instances | Number of Attributes | attribute Selection | 1th | 2ed | 3ed | 4th | 5th | Related Channels |
| --- | --- | --- | --- | --- | --- | --- | --- | --- | --- |
| Addict vs Others | 114 | 38 | CorrelationAttributeEval | higu_11 | higu_18 | higu_7 | higu_24 | higu_4 | 6,9,4,12,2 |
|  |  |  | Principal Components | higu_32 | higu_18 | higu_20 | higu_34 | higu_16 | 16,9,10,17,8 |
|  |  |  | ChiSquaredAttributeEval |  |  |  |  |  |  |
|  |  |  | SignificanceAttributeEval |  |  |  |  |  |  |
| All | 114 | 38 | CorrelationAttributeEval | higu_9 | higu_33 | higu_7 | higu_35 | higu_3 | 5,17,4,18,2 |
|  |  |  | Principal Components | higu_32 | higu_18 | higu_20 | higu_34 | higu_16 | 16,9,10,17,8 |
|  |  |  | ChiSquaredAttributeEval |  |  |  |  |  |  |
|  |  |  | SignificanceAttributeEval |  |  |  |  |  |  |
| First vs Addict | 50 | 38 | CorrelationAttributeEval | higu_7 | higu_29 | higu_33 | higu_37 | higu_11 | 4,15,17,19,6 |
|  |  |  | Principal Components | higu_20 | higu_32 | higu_18 | higu_12 | higu_2 | 10,16,9,6,1 |
|  |  |  | ChiSquaredAttributeEval |  |  |  |  |  |  |
|  |  |  | SignificanceAttributeEval |  |  |  |  |  |  |
| First vs Others | 114 | 38 | CorrelationAttributeEval | higu_33 | higu_7 | higu_29 | higu_9 | higu_37 | 17,4,15,5,19 |
|  |  |  | Principal Components | higu_32 | higu_18 | higu_20 | higu_34 | higu_16. | 16,9,10,17,8 |
|  |  |  | ChiSquaredAttributeEval | higu_29 |  |  |  |  | 15 |
|  |  |  | SignificanceAttributeEval | higu_29 |  |  |  |  | 15 |
| First vs Second | 60 | 38 | CorrelationAttributeEval | higu_9 | higu_7 | higu_33 | higu_31 | higu_3 | 5,4,17,16,2 |
|  |  |  | Principal Components | higu_6 | higu_12 | higu_25 | higu_37 | higu_10 | 3,6,13,19,5 |
|  |  |  | ChiSquaredAttributeEval |  |  |  |  |  |  |
|  |  |  | CorrelationAttributeEval |  |  |  |  |  |  |
| Normal vs Addict | 54 | 38 | CorrelationAttributeEval | higu_11 | higu_21 | higu_25 | higu_35 | higu_24 | 6,11,13,18,12 |
|  |  |  | Principal Components | higu_18 | higu_17 | higu_11 | higu_34 | higu_3 | 9,9,6,17,2 |
|  |  |  | ChiSquaredAttributeEval |  |  |  |  |  |  |
|  |  |  | SignificanceAttributeEval |  |  |  |  |  |  |
| Normal vs First | 66 | 38 | CorrelationAttributeEval | higu_33 | higu_7 | higu_29 | higu_8 | higu_5 | 17,4,15,4,3 |
|  |  |  | Principal Components | higu_21 | higu_9 | higu_37 | higu_17 | higu_7 | 11,5,19,9,4 |
|  |  |  | ChiSquaredAttributeEval |  |  |  |  |  |  |
|  |  |  | SignificanceAttributeEval |  |  |  |  |  |  |
| Normal vs Others | 114 | 38 | CorrelationAttributeEval | higu_8 | higu_35 | higu_19 | higu_21 | higu_34 | 4,18,10,11,17 |
|  |  |  | Principal Components | higu_32 | higu_18 | higu_20 | higu_34 | higu_16 | 16,9,10,17,8 |
|  |  |  | ChiSquaredAttributeEval |  |  |  |  |  |  |
|  |  |  | SignificanceAttributeEval |  |  |  |  |  |  |
| Normal vs Second | 64 | 38 | CorrelationAttributeEval | higu_9 | higu_27 | higu_35 | higu_3 | higu_5 | 5,14,18,2,3 |
|  |  |  | Principal Components | higu_21 | higu_7 | higu_15 | higu_3 | higu_17 | 11,4,8,2,9 |
|  |  |  | ChiSquaredAttributeEval | higu_9 |  |  |  |  | 5 |
|  |  |  | SignificanceAttributeEval | higu_9 |  |  |  |  | 5 |
| Second vs Addict | 48 | 38 | CorrelationAttributeEval | higu_18 | higu_10 | higu_4 | higu_24 | higu_6 | 9,5,2,12,3 |
|  |  |  | Principal Components | higu_32 | higu_20 | higu_16 | higu_8 | higu_17 | 16,10,8,4,17 |
|  |  |  | ChiSquaredAttributeEval |  |  |  |  |  |  |
|  |  |  | SignificanceAttributeEval |  |  |  |  |  |  |
| Second vs Others | 114 | 38 | CorrelationAttributeEval | higu_9 | higu_27 | higu_5 | higu_35 | higu_3 | 5,14,3,18,2 |
|  |  |  | Principal Components | higu_32 | higu_18 | higu_20 | higu_34 | higu_16 | 16,9,10,17,8 |
|  |  |  | ChiSquaredAttributeEval |  |  |  |  |  |  |
|  |  |  | SignificanceAttributeEval |  |  |  |  |  |  |

**Table S3**: Detailed comparison of the variables that show a statistically significant difference between the two groups.

| Variable | Related channel | Group 1 | Group 2 | P value |
| --- | --- | --- | --- | --- |
| higu_7 | 4 | Addict | first | 0.006 |
| higu_9 | 5 | first | second | 0.038 |
| higu_11 | 6 | Addict | first | 0.027 |
| higu_17 | 9 | Addict | first | 0.052 |
| higu_29 | 15 | Addict | first | 0.017 |
| higu_33 | 17 | Addict | first | 0.014 |
| higu_33 | 17 | first | second | 0.053 |
| higu_37 | 19 | Addict | first | 0.054 |
